# Experimental Study on Surface Integrity of Nickel-Based Superalloy in Ultrasonic Elliptical Vibration Cutting

**DOI:** 10.3390/mi16070728

**Published:** 2025-06-22

**Authors:** Gaofeng Hu, Yanjie Lu, Shengming Zhou, Min Zhang, Xin He, Fenghui Zhang, Guangjun Chen

**Affiliations:** 1 College of Mechanical Engineering, Tianjin University of Technology and Education, Tianjin 300222, China; 17860385863@163.com (Y.L.); 0322231061@tute.edu.cn (S.Z.); 0322221082@tute.edu.cn (M.Z.); zfh0476@126.com (F.Z.); chenguangjun@126.com (G.C.); 2 Tianjin Key Laboratory of High Performance Manufacturing Technology & Equipment, Tianjin 300222, China; 3 Engineering Training Center, Tianjin University of Technology and Education, Tianjin 300222, China; kernel139@163.com

**Keywords:** ultrasonic elliptical vibration cutting, nickel-based superalloys, surface integrity

## Abstract

Nickel-based superalloys, renowned for their exceptional high-temperature strength, oxidation resistance, and corrosion resistance, have become essential materials in the aerospace, defense, and nuclear industries. However, due to their poor machinability, common cutting processes often result in poor surface quality, difficulties in chip breaking, and significant tool wear. This study investigates the surface integrity of nickel-based superalloys during ultrasonic elliptical vibration cutting. The effects of various process parameters on the surface roughness, residual stress, and microhardness are systematically analyzed. The results indicate that under ultrasonic elliptical vibration cutting conditions, the surface roughness of the workpiece increases with the ultrasonic amplitude, cutting depth, and feed rate. It initially decreases and then increases with cutting speed, and decreases with an increase in the tool tip radius. The post-cutting residual stress in the nickel-based superalloy decreases with higher cutting speed and ultrasonic amplitude, but increases with greater cutting depth and tool tip radius. The surface microhardness increases with the cutting speed up to a point, after which it decreases, while it significantly increases with a higher ultrasonic amplitude, feed rate, and cutting depth. A comparative experiment was conducted between ultrasonic elliptical vibration and conventional cutting. The research results showed that when the cutting depth was 2 µm, the surface roughness and wear decreased by 19% and 53%, respectively, and the residual compressive stress and microhardness increased by 44% and 21%, respectively. This further verified the significant advantages of ultrasonic elliptical vibration cutting in optimizing machining performance.

## 1. Introduction

With the rapid advancement of science and technology, increasingly stringent demands are being placed on the mechanical properties, surface quality, cutting forces, and tool wear of materials across various global industries [1]. Many hard-to-machine materials and composites, such as optical glass, ceramics, and titanium alloys, have found widespread application in machining processes. In particular, in the aerospace industry, these difficult-to-machine materials have become the preferred choice for manufacturing key components such as turbine discs [2], casings, compressor discs, and engine brackets [3]. Nickel-based superalloys, known for their excellent high-temperature strength, oxidation resistance [4], and corrosion resistance, are well-suited to meet the demands for high-strength components in high-temperature environments [5], such as those found in aerospace. As a result, they have found widespread application in high-end industries, including aerospace [6], defense, petrochemicals, and nuclear power [7].

However, in the precision machining process of nickel-based superalloys, problems such as severe tool wear, poor surface machining quality, and difficulty in chip breaking often occur. Traditional cutting methods have proven insufficient to address the various challenges associated with machining nickel-based superalloys. In recent years, with the rise of composite machining technology, ultrasonic elliptical vibration cutting has shown unique advantages, greatly improving the cutting performance of nickel-based high-temperature alloys and demonstrating enormous application potential.

In terms of the surface morphology during the processing of difficult-to-machine materials, Kang et al. [8] compared the surface morphology of tungsten alloys processed by traditional cutting and UEVC, finding that UEVC reduces surface defects and improves the surface roughness. Zhou Haoyan from Guangdong University of Technology [9] conducted cutting tests on 304 stainless steel and found that UEVC yields better results at low cutting speeds, smaller cutting depths, and lower feed rates, making it more suitable for finishing operations. Su et al. [10] combined longitudinal and bending vibrations to design an eccentric conical ultrasonic elliptical vibration cutting tool, followed by cutting experiments. The results demonstrated that the newly developed eccentric conical UEVC tool improved the surface quality and reduced tool wear. Wu et al. [11] conducted single-factor cutting experiments on nickel-based superalloys using ceramic tools. Through systematic experiments and data analysis, they found that among the various factors affecting the surface roughness, the cutting parameters ranked in importance as follows: feed rate, cutting speed, and cutting depth. Liu Ting [12] conducted orthogonal experiments on GH4169 using Sialon ceramic inserts and performed a range analysis on the surface roughness results. She found that the factors influencing surface roughness, ranked by importance, are as follows: feed rate, cooling conditions, cutting speed, and cutting depth. Ning et al. [13] emphasized that the surface morphology is crucial for assessing the machining quality and component performance. A novel modeling approach was developed to predict the surface morphology of ultra-precision single-point diamond turning (SPDT) by considering the planned tool path. Its effectiveness was validated through simulation and experimental verification.

Residual stress is an important factor affecting the surface integrity of nickel-based superalloys. Arunachalam et al. [3] studied the impact of CBN and hybrid ceramic tools on residual stress in planar machining, finding that CBN tools generate compressive residual stress at cutting speeds of 150 m/min and 225 m/min. Yao Changfeng et al. [14] investigated the effects of tool wear on cutting forces and shear zone temperature through GH4169 turning experiments and Deform-3D simulations. They found that tool wear significantly increases the cutting forces and shear zone temperature, leading to higher peak residual tensile and compressive stresses as well as a greater depth of the residual stress layer, which substantially affects component performance. Madariaga et al. [15] studied the effect of the tool tip radius on the surface residual stress of Inconel 718 under cutting speeds of 30–70 m/min and feed rates of 0.15–0.25 mm/rev. The study found that an increase in the tool tip radius leads to an increase in the residual compressive stress. Sharman et al. [16] further investigated the influence of the tool tip radius and feed rate on residual stress. The results indicated that an increase in the feed rate leads to larger residual tensile stresses. Du Hao [17] used finite element simulation software to model the turning process of GH4169, revealing that the feed rate has the greatest influence on residual stress. Ni et al. [18] discussed the cutting performance of Ti-6Al-4V alloy formed by SLM, including the cutting force, surface morphology and roughness, subsurface microstructure evolution, residual stress changes, and tool wear characteristics.

R.S. Pawade et al. [19] conducted a comprehensive study on the deformation beneath the machined surface during nickel-based superalloy turning. Using residual stress, microhardness, and work hardening as criteria, they identified the optimal machining conditions for surface integrity. Sun Shilei et al. [20] focused on the high-speed milling of GH4169, thoroughly analyzing the changes in work hardening. Through a series of experiments, they found that the hardening degree of the machined surface layer fluctuated between 110% and 128%. In practical production, these findings can guide the rational selection of cutting parameters, balancing work hardening control, machining efficiency, and tool life.

This article systematically investigates the effect of ultrasonic elliptical vibration cutting on the surface integrity of nickel-based superalloys. The remainder of this paper is organized as follows: Section 2 analyzes the mechanism of ultrasonic elliptical vibration cutting, laying the foundation for experimental research on the surface integrity of nickel-based superalloys under ultrasonic elliptical vibration cutting; Section 3 describes the experimental research conducted to analyze the influence of ultrasonic elliptical vibration cutting on key performance indicators such as the surface roughness, surface morphology, residual stress, and surface microhardness of nickel-based superalloys under different process parameters. Section 4 compares the effects of ultrasonic elliptical vibration cutting and conventional cutting machining. Section 5 summarizes the findings of this paper.

## 2. The Mechanism of Ultrasonic Elliptical Vibration Cutting

**Figure 1:**
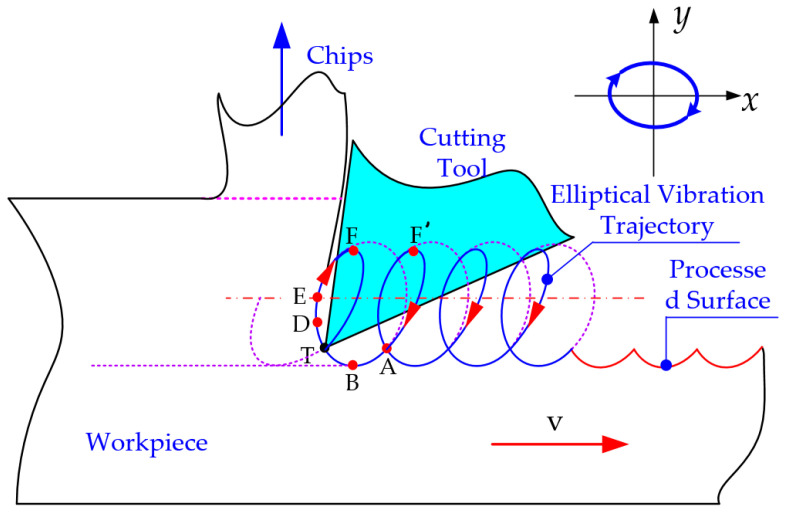
Ultrasonic elliptic vibration cutting mechanism diagram.

The mechanism of ultrasonic elliptical vibration cutting involves applying ultrasonic vibrations with different amplitudes and a 90° phase difference in the cutting and cutting depth directions. This results in the tool moving along an elliptical trajectory during the machining process, as illustrated in Figure 1.

As shown in Figure 1, the elliptical trajectory of ultrasonic elliptical vibration is formed by introducing high-frequency sinusoidal vibrations in two orthogonal directions on the tool or workpiece. These directions are typically the X direction (cutting speed direction) and Y direction (chip flow direction). By superimposing the vibrations in both directions, the tool’s motion trajectory becomes elliptical due to the presence of amplitude and phase differences. The elliptical trajectory is represented as shown in Equation (1):

(1) $$\left\{\begin{array}{l} x(t)=A\sin(2\pi \mathrm{ft})\\ y(t)=B\sin(2\pi \mathrm{ft}+\varphi ) \end{array}\right.$$

The tool’s motion trajectory relative to the workpiece can be described by the following Equation (2):

(2) $$\left\{\begin{array}{l} x(t)=A\sin(2\pi \mathrm{ft})+\mathrm{vt}\\ y(t)=B\sin(2\pi \mathrm{ft}+\varphi ) \end{array}\right.$$

In this equation, **A** and **B** represent the ultrasonic amplitudes of the tool in the X and Y directions, respectively; **f** denotes the ultrasonic vibration frequency; **φ** represents the phase difference between the two sinusoidal vibration signals, and **v** is the cutting speed. By differentiating the above equation, the tool’s velocity relative to the workpiece can be obtained, as shown in Equation (3):

(3) $$\left\{\begin{array}{l} v_{x}(t)=2\pi fA\sin(2\pi \mathrm{ft})+v\\ v_{y}(t)=2\pi fB\sin(2\pi \mathrm{ft}+\varphi ) \end{array}\right.$$

Ultrasonic elliptical vibration cutting technology is classified into two processing methods, separated and non-separated, based on the vibration mode. During the cutting process, the cutting speed ratio, k [21], serves as a key factor for distinguishing between these two methods. It is defined as follows in Equation (4):

(4) $$k=\frac{v}{v_{\max}}=\frac{v}{2\pi \mathrm{fA}}$$

## 3. The Surface Integrity Experiment of Ultrasonic Elliptical Vibration Cutting for Nickel-Based Superalloys

### 3.1. Experimental Equipment

**Figure 2:**
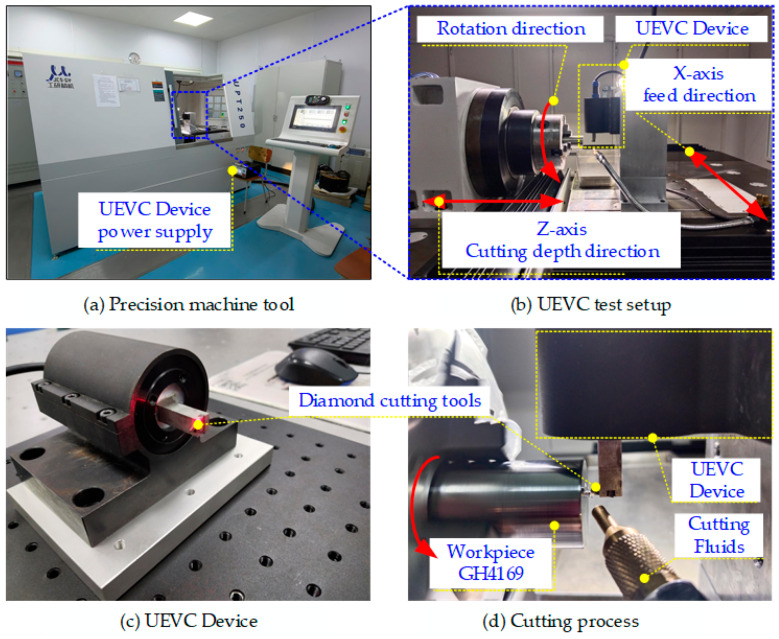
Experimental equipment.

The experiment was conducted on a UPT250 ultra-precision lathe, as shown in Figure 2b. The lathe has a maximum workpiece length of 100 mm, a maximum workpiece rotational diameter of 50 mm, and a maximum spindle speed of 3000 r/min. During the cutting process, a single-excitation ultrasonic elliptical vibration cutting device, shown in Figure 2d, was used. This device was mounted on the *X*-axis of the ultra-precision lathe and connected to an ultrasonic power supply, enabling the tool to perform ultrasonic elliptical vibration cutting. The device operates at a frequency of 41 KHz. The workpiece was clamped onto the machine spindle using a three-jaw chuck and subjected to end-face cutting. During machining, a spray-type cutting fluid was continuously applied to the contact area between the tool and the workpiece surface to ensure adequate lubrication.

### 3.2. Experimental Materials

The workpiece material used in this turning experiment was GH4169, with a workpiece size of 30 mm × 50 mm in cylindrical form. The chemical composition and key mechanical properties of the GH4169 material are listed in Table 1 and Table 2, respectively.

### 3.3. Experimental Plan

This study investigates the effects of cutting speed, cutting depth, feed rate, tool tip radius, and the presence of ultrasonic elliptical vibration on the surface integrity of a nickel-based superalloy during cutting. The experimental design, based on a single-factor approach, is outlined in Table 3. The objective is to examine how varying cutting parameters influence surface integrity metrics during ultrasonic elliptical vibration cutting of the superalloy. Additionally, a comparative analysis is performed to evaluate the differences between conventional cutting and ultrasonic elliptical vibration cutting processes.

### 3.4. Surface Integrity Testing Methods

**Figure 3:**
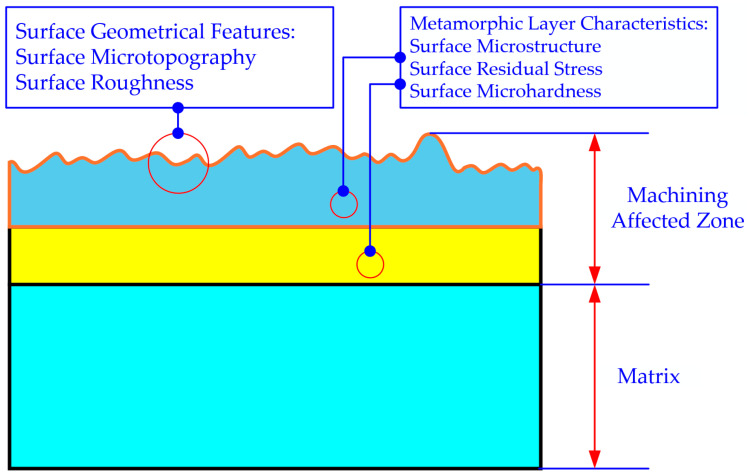
Study indicators for surface integrity of GH4169 in ultrasonic elliptical vibration cutting.

This study selected surface morphology, surface roughness, surface residual stress, and surface microhardness as the key indicators, as shown in the figure, to investigate the surface integrity of nickel-based superalloy during ultrasonic elliptical vibration cutting. A comprehensive analysis of these key performance parameters was conducted to explore the variations in surface quality under different cutting conditions, as illustrated in Figure 3.

**Figure 4:**
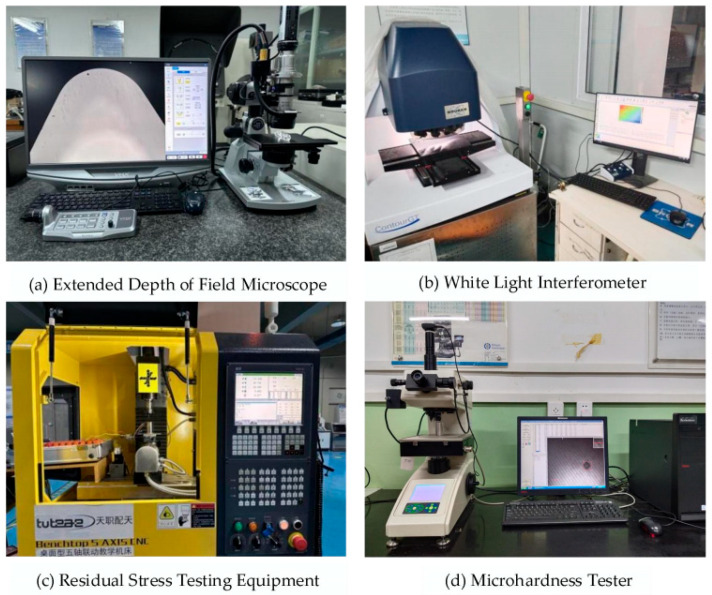
Surface integrity testing equipment.

To investigate the effects of different experimental parameters on the surface morphology, a precise measurement of the workpiece surface and surface defects was performed using a VHX970F super-depth 3D microscope (KEYENCE, Osaka, Japan), as illustrated in Figure 4a. The surface was further analyzed using the ContourGT white light interferometer, manufactured by Bruker (Bruker Corporation, Billerica, MA, USA), which allows for effective observation of the three-dimensional surface morphology and precise measurement of surface roughness values, as illustrated in Figure 4b. Residual stress measurements on the processed surface were carried out using the blind hole method, employing a desktop five-axis machine tool and a strain gauge, as illustrated in Figure 4c. For accurate hardness measurement of the workpiece after ultrasonic elliptical vibration cutting, a Wilson Vickers microhardness tester was utilized, as illustrated in Figure 4d.

### 3.5. Analysis of Surface Roughness in Ultrasonic Elliptical Vibration Cutting

#### 3.5.1. The Effect of Cutting Speed on Surface Roughness

**Figure 5:**
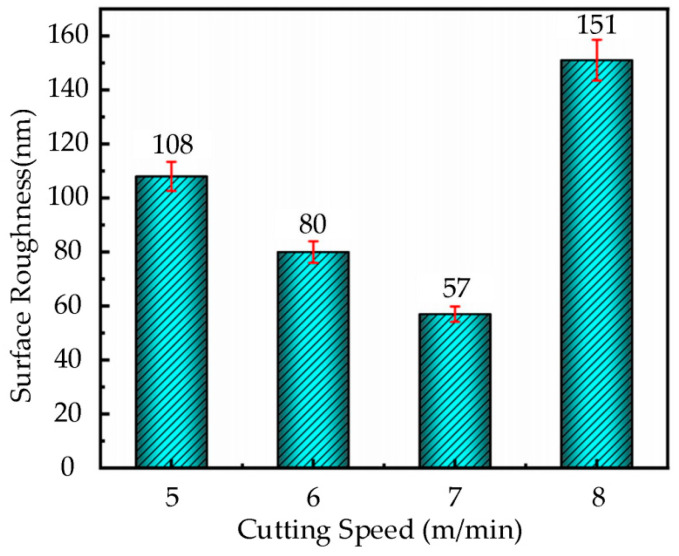
Histogram of surface roughness change with cutting speed in UEVC.

Based on the cutting parameters provided in Table 3, a diamond tool with a feed rate of 22 µm/rev, cutting depth of 2 µm, ultrasonic amplitudes of **A** = 7.0 µm and **B** = 1.7 µm, and a tool tip radius of 0.5 mm was used in this experiment. The ultrasonic elliptical vibration cutting experiments were conducted on the nickel-based superalloy workpiece under cutting speeds of 5 m/min, 6 m/min, 7 m/min, and 8 m/min. The bar chart in Figure 5 shows the surface roughness of the workpiece at different cutting speeds.

As shown in Figure 5, the surface roughness initially decreases and then increases as the cutting speed increases. Specifically, when the cutting speed increases from 5 m/min to 7 m/min, the surface roughness gradually decreases, indicating an improvement in the cutting performance within this speed range. However, when the cutting speed reaches 8 m/min, the surface roughness sharply increases, leading to a significant deterioration in surface quality. Therefore, selecting an appropriate cutting speed is critical when performing ultrasonic elliptical vibration cutting on nickel-based superalloys. Maintaining the cutting speed below 7 m/min can effectively control surface roughness and ensure a more optimal surface quality.

#### 3.5.2. The Effect of Feed Rate on Surface Roughness

**Figure 6:**
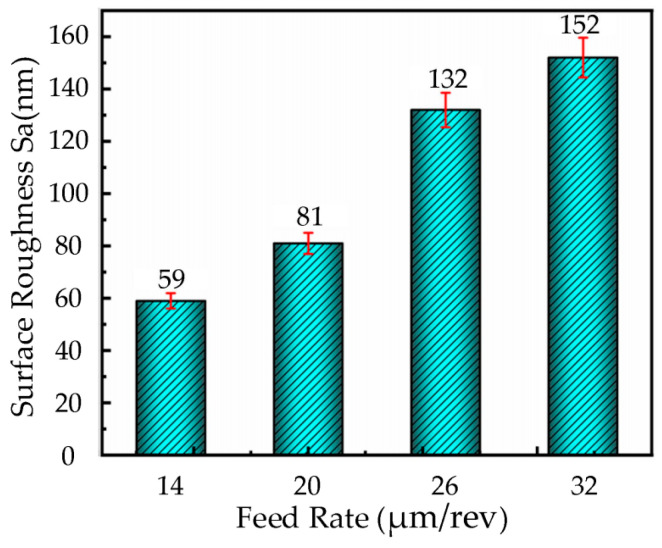
Histogram of surface roughness change with feed rate in UEVC.

Based on the cutting parameters provided in Table 3, ultrasonic elliptical vibration cutting experiments were conducted on nickel-based superalloy workpieces using a diamond tool with a cutting speed of 6 m/min, cutting depth of 3.5 µm, ultrasonic amplitudes of **A** = 7.0 µm and **B** = 1.7 µm, and a tool nose radius of 0.5 mm. The feed rates were set at 14 µm/rev, 20 µm/rev, 26 µm/rev, and 32 µm/rev. The bar chart in Figure 6 illustrates the surface roughness of the workpieces at different feed rates.

As shown in Figure 6, the surface roughness of the workpieces increases significantly with the feed rate. Specifically, when the feed rate is 14 µm/rev, the surface roughness is 59 nm. However, when the feed rate is increased to 32 µm/rev, the surface roughness sharply rises to 152 nm, a 2.6-fold increase, indicating that the feed rate has a significant impact on surface roughness. Further analysis reveals that at lower feed rates, the tool’s feed distance per unit of time is shorter, resulting in a higher overlap between adjacent tool marks and a more uniform, continuous surface texture, thereby significantly reducing surface roughness. On the other hand, when the feed rate is too high, the tool’s movement distance increases, reducing the overlap between adjacent tool marks, which leaves distinct tool marks on the surface and significantly increases the roughness. Therefore, selecting an appropriate feed rate is crucial for achieving optimal surface quality.

#### 3.5.3. The Effect of Cutting Depth on Surface Roughness

**Figure 7:**
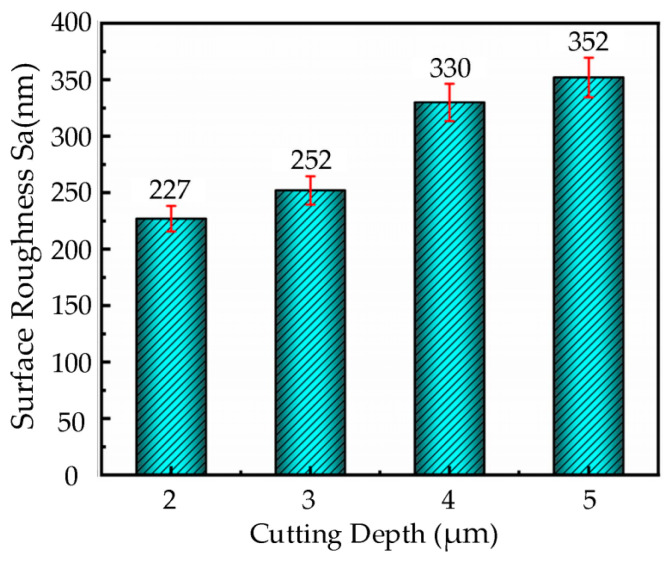
Histogram of surface roughness change with cutting depth in UEVC.

Based on the cutting parameters provided in Table 3, ultrasonic elliptical vibration cutting experiments were conducted on the nickel-based superalloy workpieces using a diamond tool with a cutting speed of 7 m/min, feed rate of 36 µm/rev, ultrasonic amplitudes of **A** = 7.0 µm and **B** = 1.7 µm, and a tool tip radius of 0.5 mm. The experiments were performed at different cutting depths of 2 µm, 3 µm, 4 µm, and 5 µm. The resulting surface roughness of the workpieces at these depths is presented in the bar chart shown in Figure 7.

From the bar chart in Figure 7, it can be observed that the surface roughness of the workpiece gradually increases with the cutting depth. When the cutting depth is increased from 2 µm to 4 µm, the surface roughness increases, but the change is relatively mild. This may be due to the fact that within this depth range, the increase in the cutting layer thickness does not significantly affect the interaction between the cutting tool and the material. However, when the cutting depth is increased from 4 µm to 5 µm, a significant increase in surface roughness is observed, indicating that the larger cutting depth exceeds the optimized effect of ultrasonic vibration cutting. This leads to reduced material removal efficiency, increased cutting forces, and heat accumulation, which significantly deteriorates surface quality.

#### 3.5.4. Effect of Ultrasonic Amplitude on Surface Roughness

**Figure 8:**
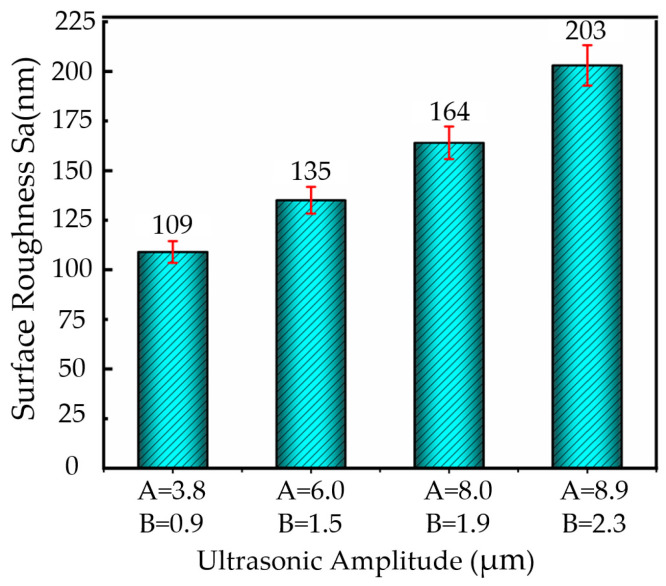
Surface roughness changes with ultrasonic amplitude in UEVC.

Based on the experimental cutting parameters, a diamond tool with a cutting speed of 8 m/min, feed rate of 36 µm/rev, cutting depth of 2 µm, and a nose radius of 0.5 mm was used, combined with different ultrasonic amplitude conditions (**A** = 3.8 µm, **B** = 0.9 µm; **A** = 6.0 µm, **B** = 1.5 µm; **A** = 8.0 µm, **B** = 1.9 µm; **A** = 8.9 µm, **B** = 2.3 µm). Ultrasonic elliptical vibration cutting experiments were conducted on nickel-based superalloy workpieces. Figure 8 presents the bar chart of surface roughness for the workpieces under different ultrasonic amplitude conditions.

As shown in Figure 8, with the increase in the ultrasonic amplitude in both directions, the surface roughness of the workpiece also gradually increases. When the ultrasonic amplitude is **A** = 3.9 µm and **B** = 0.9 µm, the surface roughness is minimized, to approximately 109 nm. However, when the ultrasonic amplitude increases to **A** = 8.9 µm and **B** = 2.3 µm, the surface roughness sharply rises to 203 nm, exhibiting a significant increase.

#### 3.5.5. The Effect of Tool Nose Radius on Surface Roughness

**Figure 9:**
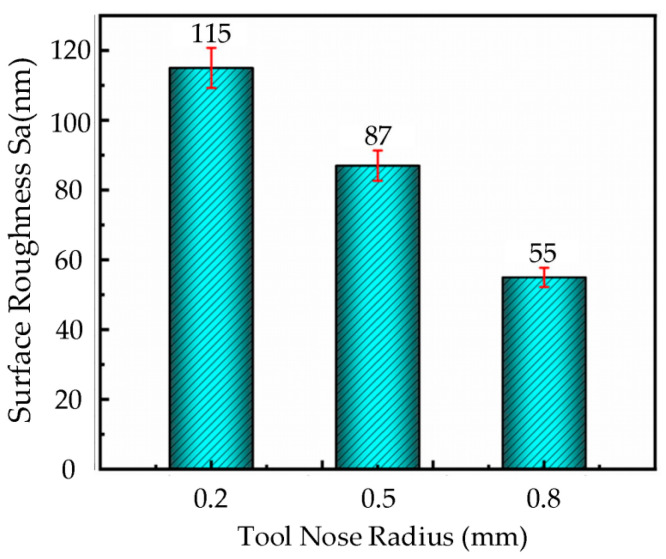
Histogram of surface roughness change with tool nose radius in UEVC.

Based on the given cutting parameters, ultrasound elliptical vibration cutting experiments were conducted on the nickel-based superalloy workpiece using diamond tools with different tool nose radii (0.2 mm, 0.5 mm and 0.8 mm) at a cutting speed of 6 m/min, feed rate of 18 µm/rev, cutting depth of 2.5 µm, and ultrasonic amplitude of **A** = 7.0 µm, **B** = 1.7 µm. Figure 9 presents the bar chart of surface roughness under different tool nose radius conditions.

As shown in Figure 9, the surface roughness of the workpiece significantly decreases with the increase in tool nose radius. When the tool nose radius is 0.8 mm, the workpiece surface exhibits a relatively smooth morphology, with a surface roughness of only 63 nm. In contrast, when the tool nose radius is 0.2 mm, noticeable defects appear on the surface, resulting in a higher surface roughness of 115 nm.

### 3.6. Analysis of Surface Residual Stress in Ultrasonic Elliptical Vibration Cutting

#### 3.6.1. Effect of Cutting Speed on Residual Stress

**Figure 10:**
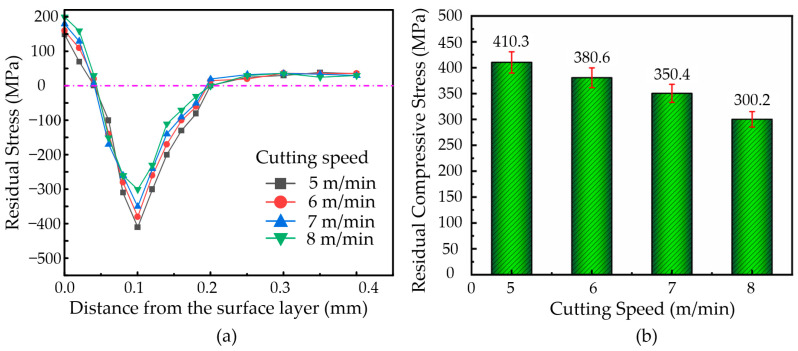
(**a**) Variation of residual stress with depth along the surface. (**b**) Variation in residual compressive stress with different cutting speeds.

During the machining of the nickel-based superalloys, an ultrasonic amplitude set at **A** = 7.0 µm and **B** = 1.7 µm, a vibration frequency of 41 KHz, a cutting depth of **a_p_** = 3 µm, a feed rate of **f** = 36 µm/rev, and cutting speeds of 5 m/min, 6 m/min, 7 m/min, and 8 m/min were selected. The variation in residual stress with depth at different cutting speeds, as well as the change in the residual compressive stress with the cutting speed, were plotted, as shown in Figure 10.

As shown in Figure 10a, the variation trend of residual stress with depth is similar at different cutting speeds. Specifically, within the depth range of 0–0.1 mm, the change in residual stress is relatively large. In the 0.1–0.2 mm depth range, the variation in residual stress decreases, and when the depth exceeds 0.2 mm, the residual stress approaches zero. According to Figure 10b, the surface of the nickel-based superalloy material is primarily under residual tensile stress, while the internal residual stress is predominantly compressive. As the cutting speed increases, the maximum residual stress within the material gradually decreases. At a cutting speed of 5 m/min, the internal residual compressive stress is higher, at 410.3 MPa.

Higher cutting speeds result in increased material removal, which also raises the cutting temperature. The cutting temperature has a greater influence on the residual stress of the machined surface and may lead to the formation of residual tensile stress on the surface. Within the cutting speed range of 5–8 m/min, the residual tensile stress gradually increases as the cutting speed rises. The low thermal conductivity of nickel-based superalloy materials makes it difficult for heat to effectively dissipate into the material’s interior. Therefore, the cutting force has a more significant impact on the internal material, and as the cutting speed increases, the cutting force decreases, leading to a reduction in the internal residual compressive stress.

#### 3.6.2. The Effect of Cutting Depth on Residual Stress

**Figure 11:**
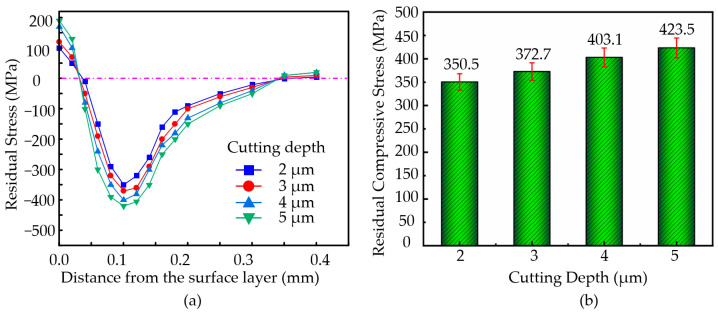
(**a**) Variation in residual stress with depth along the surface. (**b**) Variation in residual compressive stress with different cutting depths.

During the machining of the nickel-based superalloys, the ultrasonic amplitude was set to **A** = 7.0 µm and **B** = 1.7 µm, with a vibration frequency of 41 KHz, a cutting speed of **v** = 7 m/min, and a feed rate of **a_p_** = 36 µm/rev. The cutting depths were set to 2 µm, 3 µm, 4 µm, and 5 µm. Based on experimental data obtained at different cutting depths, the variation curves of residual stress with depth and the bar chart of residual compressive stress with cutting depth were plotted, as shown in Figure 11.

As shown in Figure 11a, the variation trends of residual stress with depth are generally consistent across different cutting depths. Within the depth range of 0–0.1 mm, the change in residual stress is more significant. In the 0.1–0.2 mm range, the variation is smaller, and beyond 0.2 mm, the residual stress value approaches zero. As depicted in Figure 11b, with an increase in the cutting depth, the surface residual tensile stress continuously increases. Simultaneously, the residual compressive stress inside the material also increases with the cutting depth. When the cutting depth reaches **a_p_** = 5 µm, the maximum residual stress inside the workpiece reaches 423.5 MPa.

As the cutting depth increases, cutting forces significantly rise, intensifying the mechanical pressure exerted by the tool on the workpiece, leading to larger residual compressive stresses on the surface and subsurface, with a deeper distribution. However, an increase in the cutting depth also raises the cutting temperatures, amplifying thermal expansion and cooling contraction effects, which may reduce the magnitude of residual compressive stress. At smaller cutting depths, lower cutting forces and predominant thermal effects result in more noticeable surface residual tensile stress. Moderate cutting depths strike a balance between mechanical effects and thermal effects, generating larger residual compressive stresses. Conversely, excessively large cutting depths may lead to increased surface roughness and stress concentration.

#### 3.6.3. The Effect of Ultrasonic Amplitude on Residual Stress

**Figure 12:**
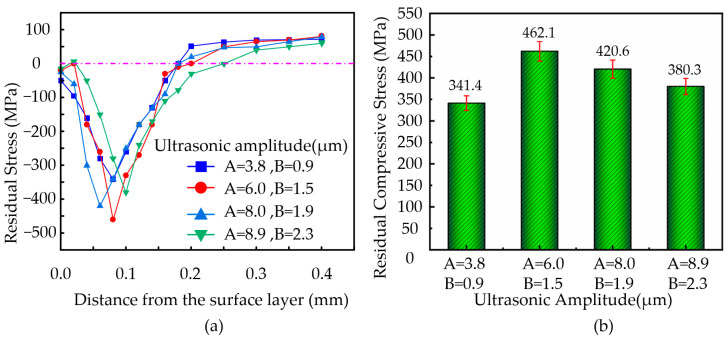
(**a**) Variation of residual stress with depth along the surface. (**b**) Variation in residual compressive stress with different ultrasonic amplitudes.

During the machining of nickel-based superalloys, the vibration frequency was set to 41 KHz, the cutting speed to 8 m/min, the feed rate to 36 µm/rev, and the cutting depth to 2 µm. The ultrasonic amplitudes were set at **A** = 3.8 µm, **B** = 0.9 µm; **A** = 6.0 µm, **B** = 1.5 µm; **A** = 8.0 µm, **B** = 1.9 µm; and **A** = 8.9 µm, **B** = 2.3 µm. The variation in residual stress with depth at the workpiece surface and the change in residual compressive stress with ultrasonic amplitude were plotted, as shown in Figure 12.

As shown in Figure 12a, the trend in residual stress variation with depth is similar under different ultrasonic amplitudes. Within the depth range of 0–0.1 mm, the residual stress exhibits significant changes. In the 0.1–0.2 mm depth range, the variation in residual stress is smaller. When the depth exceeds 0.2 mm, the residual stress approaches zero. Figure 12b illustrates that as the ultrasonic amplitude increases, the residual tensile stress at the workpiece surface continuously increases, while the residual compressive stress inside the material decreases with higher ultrasonic amplitudes. When the ultrasonic amplitude is set to **A** = 6.0 µm, **B** = 1.5 µm, the maximum residual compressive stress inside the material reaches 462.1 MPa.

As the ultrasonic amplitude increases, both the magnitude and the distribution range of residual compressive stress decrease. This is due to the enhanced contact–separation effect between the tool and the workpiece, which weakens the mechanical squeezing effect, thereby reducing plastic deformation. However, if the amplitude is too high, the accumulation of cutting heat significantly increases, further diminishing the effect of compressive stress and potentially inducing residual tensile stress at the surface.

#### 3.6.4. The Effect of Tool Tip Radius on Residual Stress

During the machining of the nickel-based superalloys, with an ultrasonic amplitude of **A** = 7.0 µm, **B** = 1.7 µm, vibration frequency of 41 KHz, cutting speed of **v** = 5 m/min, feed rate of **f** = 18 µm/rev, and cutting depth of **a_p_** = 2.5 µm, the tool tip radius was set to 0.2 mm, 0.5 mm, and 0.8 mm. 

**Figure 13:**
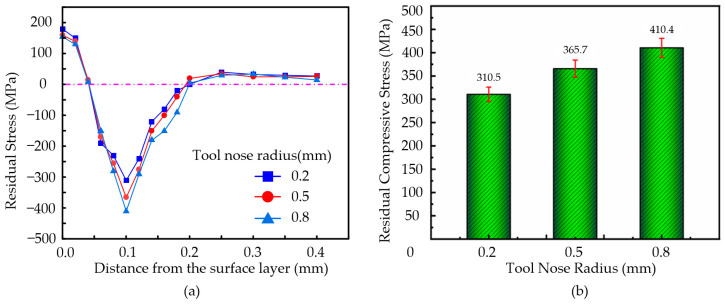
(**a**) Variation of residual stress with depth along the surface. (**b**) Variation in residual compressive stress with different tool nose radii.

The variation in the residual stress with depth of the surface layer was shown in Figure 13a and the residual compressive stress with the tool nose radius, as shown in Figure 13b.

As shown in Figure 13a, the variation in the residual stress with depth follows a similar trend under different tool nose radius. In the depth range of 0–0.1 mm, the residual stress exhibits significant variation. In the 0.1–0.2 mm depth range, the variation becomes smaller. When the depth exceeds 0.2 mm, the residual stress approaches zero. As illustrated in Figure 13b, the residual compressive stress inside the workpiece increases with the tool tip radius. When the tool nose radius is 0.8 mm, the maximum residual compressive stress within the workpiece reaches approximately 410.4 MPa.

A smaller tool nose radius introduces less residual compressive stress with a shallower distribution. However, due to the sharper cutting trajectory, this can result in increased surface roughness and potentially lead to surface defects. Conversely, a larger tool nose radius increases the contact area between the tool and the workpiece, leading to higher contact pressure. With cutting forces are concentrated over a larger area, the workpiece surface undergoes stronger mechanical compression, causing greater plastic deformation in the surface layer and resulting in higher residual compressive stress. Nevertheless, an excessively large radius may lead to increased cutting heat accumulation and friction, thereby weakening the residual compressive stress effect.

### 3.7. Analysis of Microhardness on the Surface of Ultrasonic Elliptical Vibration Cutting

#### 3.7.1. Effect of Cutting Speed on Microhardness

**Figure 14:**
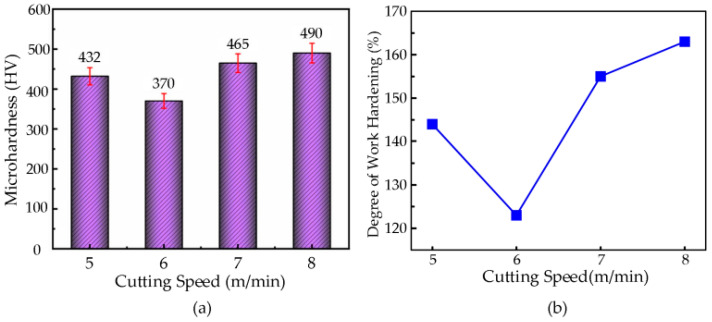
(**a**) Relationship Between cutting speed and microhardness. (**b**) Relationship curve between cutting speed and degree of work hardening.

A series of single-factor cutting experiments were conducted on a nickel-based superalloy, and the experimental data were analyzed in conjunction with microhardness testing. The variation in the microhardness at different cutting speeds is shown in Figure 14a. In the experiments, the feed rate was set at *f* = 36 µm/rev, cutting depth at **a_p_** = 3.5 µm, ultrasonic amplitude at **A** = 7.0 µm, **B** = 1.7 µm, and the cutting tool used was a diamond tool with a nose radius of **r_e_** = 0.5 mm. The cutting speeds were set at 5 m/min, 6 m/min, 7 m/min, and 8 m/min. The results showed that the microhardness of the machined surface of the nickel-based superalloy first decreased and then increased with the increase in cutting speed.

At a cutting speed of **v** = 8 m/min, the highest microhardness value of 490 HV was achieved. Conversely, at **v** = 6 m/min, the lowest microhardness value of 370 HV was observed. The increase in the cutting speed led to a reduction in the depth of the hardened layer on the machined surface, resulting in a lower microhardness. In the cutting speed range of **v** = 5–6 m/min, as the cutting speed increased, the deformation rate in the cutting zone also increased, reducing the contact time between the tool’s rake face and the workpiece material, while the friction between the tool’s cutting edge and the chip decreased. This resulted in reduced plastic deformation of the machined surface, causing a decrease in the microhardness with increasing cutting speed. However, in the cutting speed range of **v** = 7–8 m/min, as the cutting speed continued to increase, the material removal rate also increased, and the degree of workpiece deformation gradually intensified, leading to an increase in the microhardness.

The workpiece surface work hardening at cutting speeds of 5 m/min, 6 m/min, 7 m/min, and 8 m/min was calculated using a formula, as shown in Figure 14b. From the figure, it can be observed that as the cutting speed increases, the degree of work hardening on the machined surface initially decreases and then increases. At a cutting speed of *v* = 8 m/min, the maximum work hardening degree of 163% is achieved, while at **v** = 6 m/min, the minimum work hardening degree of 123% is observed.

#### 3.7.2. The Effect of Feed Rate on Microhardness

**Figure 15:**
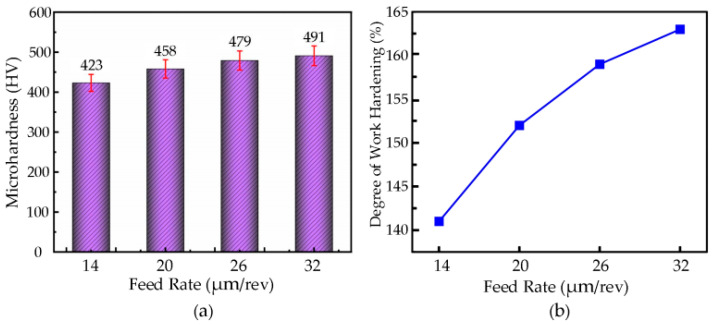
(**a**) Relationship Between feed rate and microhardness. (**b**) Relationship curve between feed rate and degree of work hardening.

The microhardness values of the workpiece were measured at different feed rates, and the experimental data were used to plot the variation in microhardness with feed rate, as shown in Figure 15a. For a cutting speed of **v** = 6 m/min, cutting depth **a_p_** = 3.5 µm, ultrasonic amplitude **A** = 7.0 µm, **B** = 1.7 µm, and tool tip radius **r_e_** = 0.5 mm, the feed rates were set to 14 µm/rev, 20 µm/rev, 26 µm/rev, and 32 µm/rev. The microhardness of the surface of the nickel-based superalloy increased with an increase in feed rate.

At a feed rate of **f** = 32 µm/rev, the highest microhardness value of 491 HV was obtained, while at a feed rate of **f** = 14 µm/rev, the lowest value of 423 HV was observed. This phenomenon can be attributed to the increased cutting force, cutting heat, and tool–workpiece contact pressure as the feed rate increases, which leads to greater deformation in the cutting zone and, subsequently, an increase in the surface microhardness with higher feed rates.

By applying the formula, the changes in the workpiece surface hardening degree at feed rates of 14 µm/rev, 20 µm/rev, 26 µm/rev, and 32 µm/rev were calculated, as shown in Figure 15b. A larger feed rate enhances the tool’s compressive and plastic deformation effects on the workpiece surface, increasing the dislocation density, which leads to a higher surface hardness and strength, while also expanding the plastic deformation zone and increasing the depth of the work-hardened layer. At a feed rate of **f** = 32 µm/rev, the maximum hardening degree of 163% was achieved, while at a feed rate of **f** = 14 µm/rev, the minimum hardening degree of 141% was observed.

#### 3.7.3. The Effect of Cutting Depth on Microhardness

**Figure 16:**
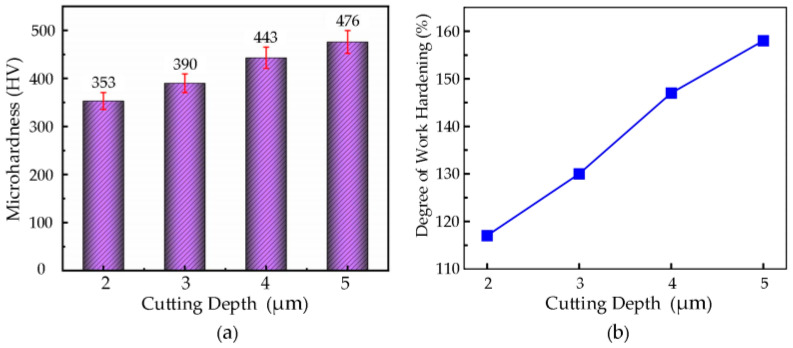
(**a**) Relationship between cutting depth and microhardness. (**b**) Relationship curve between cutting depth and degree of work hardening.

The microhardness values of the machined workpiece at different cutting depths were measured through microhardness testing. Based on the experimental data, a plot of the microhardness variation with cutting depth was generated, as shown in Figure 16a. For a cutting speed of **v** = 7 m/min, a feed rate of **f** = 36 µm/rev, ultrasonic amplitude **A** = 7.0 µm, **B** = 1.7 µm, and a tool nose radius of **r_e_** = 0.5 mm, with cutting depths of 2 µm, 3 µm, 4 µm, and 5 µm, the microhardness of the machined surface increases as the cutting depth increases.

When the cutting depth is **a_p_** = 5 µm, the maximum microhardness value of 476 HV is reached, whereas at **a_p_** = 2 µm, the minimum microhardness value is 353 HV. As the cutting depth increases, the workpiece surface and subsurface material experience greater mechanical pressure and plastic deformation, leading to a gradual increase in microhardness, with the hardened layer thickness and distribution range also increasing.

By calculating using the formula, the change in the surface workpiece hardening degree for cutting depths of 2 µm, 3 µm, 4 µm, and 5 µm was obtained, as shown in Figure 16b. As can be seen from the figure, as the cutting depth increases, the hardened degree of the machined surface also increases correspondingly. When the cutting depth is **a_p_** = 5 µm, the maximum hardening degree of the surface is 158%; when the cutting depth is **a_p_** = 2 µm, the minimum hardening degree is only 117%.

#### 3.7.4. The Effect of Ultrasonic Amplitude on Microhardness

**Figure 17:**
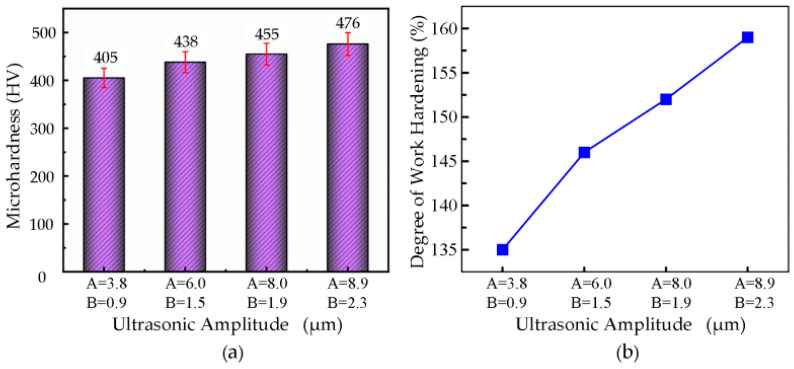
(**a**) Relationship Between ultrasound amplitude and microhardness. (**b**) Relationship curve between ultrasound amplitude and degree of work hardening.

The microhardness values of the processed workpieces were measured under different ultrasonic amplitudes through microhardness testing. The experimental data were analyzed, and a graph showing the variation in microhardness with ultrasonic amplitude was plotted, as shown in Figure 17a. In the experiments, with a cutting speed of **v** = 6 m/min, cutting depth **a_p_** = 3.5 µm, feed rate **f** = 36 µm/rev, and a tool nose radius of **r_e_** = 0.5 mm, the ultrasonic amplitudes were set at **A** = 3.8 µm, **B** = 0.9 µm; **A** = 6.0 µm, **B** = 1.5 µm; **A** = 8.0 µm, **B** = 1.9 µm; and **A** = 8.9 µm, **B** = 2.3 µm. The microhardness of the nickel-based superalloy increased with the ultrasonic amplitude.

When the ultrasonic amplitude was **A** = 8.9 µm, **B** = 2.3 µm, the highest microhardness value of 476 HV was achieved, while the lowest value of 405 HV was recorded at **A** = 3.8 µm, **B** = 0.9 µm. This phenomenon can be attributed to the fact that as the ultrasonic amplitude increases, the cutting force intensifies, resulting in a stronger rolling effect on the processed surface by the tool’s rake face, which increases the deformation in the cutting area. Consequently, the microhardness of the processed surface increases with the ultrasonic amplitude.

The changes in the workpiece surface’s degree of work hardening at ultrasonic amplitudes of **A** = 3.8 µm, **B** = 0.9 µm; **A** = 6.0 µm, **B** = 1.5 µm; **A** = 8.0 µm, **B** = 1.9 µm; and **A** = 8.9 µm, **B** = 2.3 µm were calculated using formulas, as shown in Figure 17b. The results indicate that as the ultrasonic amplitude increases, the degree of work hardening on the processed surface also increases. At **A** = 8.9 µm, **B** = 2.3 µm, the work hardening reached its maximum value of 159%, while at **A** = 3.8 µm, **B** = 0.9 µm, the work hardening was the least, at 135%.

## 4. Experiment on Surface Integrity of Ultrasonic Elliptical Vibration Cutting and Conventional Cutting

### 4.1. Comparison of Surface Roughness Between UEVC and CC

The surface morphology is composed of surface roughness, waviness, surface defects, and surface layer characteristics. Surface roughness results from the tool path and material plastic deformation, influencing the surface’s finish. Waviness is caused by machine tool vibrations and fluctuations in processing parameters, affecting assembly performance. Surface defects, such as cracks and scratches, impact the durability and fatigue resistance of the component. The surface morphology of a workpiece directly determines the machining accuracy, which, in turn, influences the final part’s functional precision. These components collectively govern the quality and performance of the machined surface.

**Figure 18:**
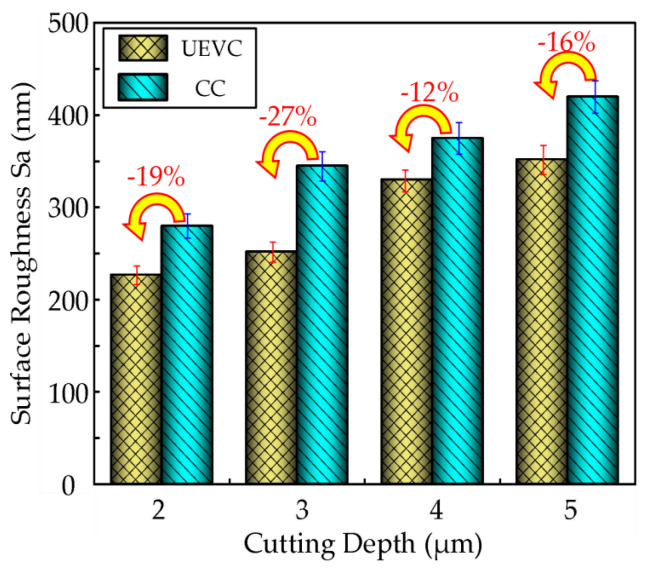
Comparison of surface roughness between UEVC and CC.

Under the same cutting parameters, as shown in Table 4, both ultrasonic elliptical vibration cutting and conventional cutting experiments were conducted on the nickel-based superalloy. The effect of the cutting depth on the surface roughness was compared, revealing similar trends for both cutting methods, with the other parameters held constant. From the bar chart in Figure 18, depicting the relationship between the cutting depth and surface roughness, it can be observed that at cutting depths of 2 µm, 3 µm, 4 µm, and 5 µm, the surface roughness with ultrasonic elliptical vibration cutting decreased by approximately 19%, 27%, 12%, and 16%, respectively, compared to that with conventional cutting. This indicates that ultrasonic elliptical vibration cutting significantly improves the surface quality.

### 4.2. Comparison of Residual Stress on Surfaces Between UEVC and CC

**Figure 19:**
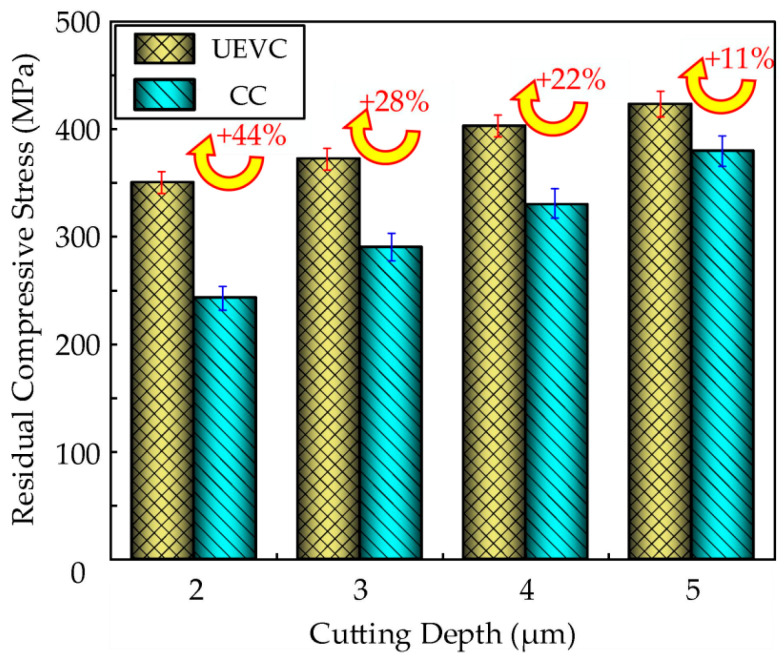
Comparison of residual compressive stress between UEVC and CC.

The variation in the residual stress of the nickel-based superalloy under both ultrasonic elliptical vibration cutting and conventional cutting methods at different cutting depths is shown in Figure 19. Significant differences in the residual compressive stress were observed between the two cutting methods. At cutting depths of 2 µm, 3 µm, 4 µm, and 5 µm, the residual compressive stress increased by approximately 44%, 28%, 22%, and 11%, respectively, under ultrasonic elliptical vibration cutting compared to conventional cutting. In conventional cutting, due to the continuous contact between the tool and the workpiece, more cutting heat accumulates, and the mechanical squeezing effect is relatively weak, resulting in lower residual compressive stress, with a shallower and more uneven stress distribution. In contrast, ultrasonic elliptical vibration cutting reduces cutting heat accumulation through high-frequency contact–separation effects, while optimizing the mechanical squeezing action. This not only introduces higher residual compressive stress but also results in a deeper and more uniform distribution of stress. This difference significantly enhances the fatigue resistance and surface quality of the workpiece, demonstrating superior machining performance under ultrasonic elliptical vibration cutting.

Under identical cutting parameters, the residual compressive stress generated in the workpiece during ultrasonic elliptical vibration cutting is typically higher than that produced by conventional cutting. This difference arises from the fact that, during ultrasonic elliptical vibration cutting, the tool not only cuts the workpiece but also applies high-frequency ultrasonic vibrations in two directions, making the cutting process intermittent. During the tool–workpiece separation phase, the high-frequency vibrations cause a reversal of the frictional forces. This reversal phenomenon facilitates the plastic flow of the surface metal, further enhancing the plastic deformation capacity of the material’s surface layer, thereby resulting in higher residual compressive stress.

### 4.3. Comparison of Surface Microhardness Between UEVC and CC

**Figure 20:**
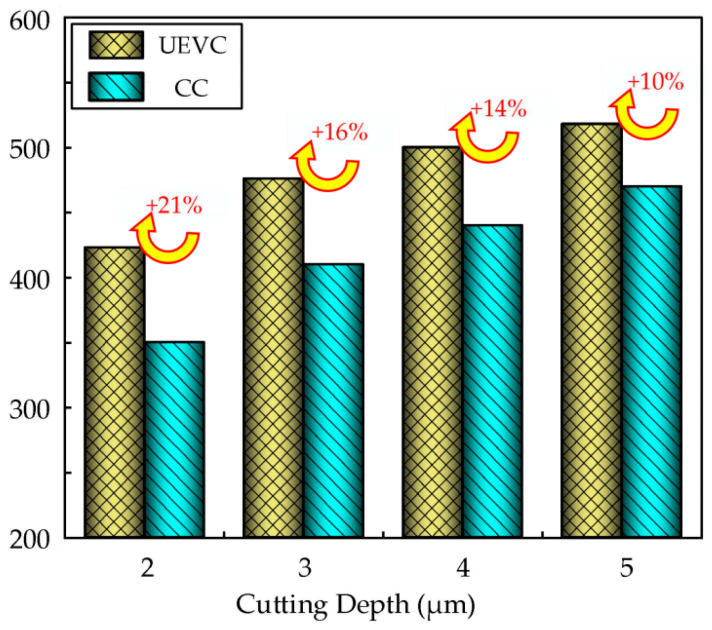
Comparison of microhardness between UEVC and CC.

Figure 20 illustrates the variation in the surface microhardness of the nickel-based superalloy workpieces under different cutting depths, comparing ultrasonic elliptical vibration cutting and conventional cutting. Both cutting methods result in an increase in the surface microhardness, exhibiting a clear work-hardening effect. At cutting depths of 2 µm, 3 µm, 4 µm, and 5 µm, the microhardness achieved by ultrasonic elliptical vibration cutting is approximately 21%, 16%, 14%, and 10% higher, respectively, than that of conventional cutting. The surface microhardness after ultrasonic elliptical vibration cutting is significantly greater than that after conventional cutting, indicating that the unique vibration effect of ultrasonic elliptical vibration cutting induces a stronger work-hardening effect on the material surface during the process.

Moreover, as the cutting depth increases, the degree of work hardening in the workpiece surface also increases, demonstrating the significant impact of the cutting depth on the work-hardening phenomenon. Under the same cutting conditions, ultrasonic elliptical vibration cutting typically results in a higher surface microhardness. This is because ultrasonic vibration improves the efficiency of tool–workpiece contact, promoting more uniform plastic deformation of the material, which enhances the hardening effect on the surface. In contrast, conventional cutting results in a more limited hardening effect with a smaller increase in surface microhardness.

In the case of ultrasonic elliptical vibration cutting, the intermittent nature of the cutting process leads to shorter contact times between the tool and workpiece, reducing the influence of cutting force and friction. This minimizes the thermal load during processing and prevents the decrease in the surface hardness caused by overheating. On the other hand, conventional cutting involves continuous cutting, with longer contact times and higher cutting forces, which can exacerbate localized overheating, resulting in a relatively lower degree of surface hardening.

### 4.4. Comparison of Chip Morphology Between UEVC and CC

**Figure 21:**
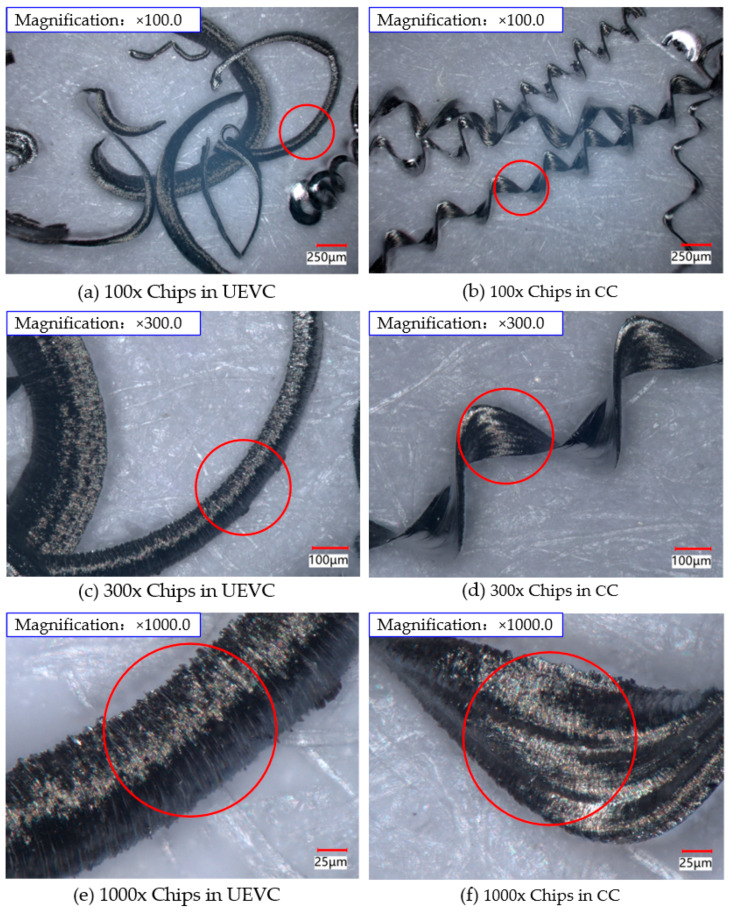
Chip morphology under **UEVC** and **CC**.

Under the conditions of ultrasonic amplitude **A** = 7.0 µm, **B** = 1.7 µm, cutting speed **v** = 5 m/min, cutting depth **a_p_** = 3 µm, and feed rate **f** = 22 µm/rev, the chip morphology during ultrasonic elliptical vibration cutting and conventional cutting was observed using a super-depth-of-field microscope system at magnifications of 100×, 300×, and 1000×. The resulting chip morphologies are shown in Figure 21.

The cutting force is a key factor affecting the chip formation process. As observed in this study, the cutting force in conventional cutting is significantly greater than that in ultrasonic elliptical vibration cutting. The larger cutting force causes an increased delamination area as the chip is sheared from the workpiece surface, which results in a wider chip cross-section. This is evident in Figure 21b,e, where the chip width in conventional cutting is noticeably larger than that produced in ultrasonic elliptical vibration cutting. Figure 21a,d illustrate that, regardless of the cutting method, the chips formed are spiral in shape, but there are distinct differences. Chips generated by ultrasonic elliptical vibration cutting are shorter, with a larger radius of curvature, resulting in a smoother cutting process. In contrast, conventional cutting produces continuous coiled chips with a greater curvature, making chip breakage more difficult. Figure 21b,c,e,f show the chip morphology at 300× and 1000× magnification for both cutting methods. It is evident that the chips from conventional cutting have irregular saw-tooth edges, with surface defects such as scratches and deep pits, which contribute to roughness in the machined surface. In comparison, the chips produced by ultrasonic elliptical vibration cutting have saw-tooth edges as well, but these are more uniform, smaller in size, with a shorter distance between the teeth, and the surface appears smoother with fewer defects.

### 4.5. Comparison of Tool Wear Between UEVC and CC

During both conventional cutting and ultrasonic elliptical vibration cutting, the tool comes into contact with the workpiece surface and removes material, resulting in varying degrees of tool wear. The extent of tool wear directly affects its lifespan. The degree of wear is closely related to the forces acting on the tool and the quality of the workpiece surface. This study uses the difficult-to-machine material, nickel-based superalloy GH4169, to analyze the impact of tool wear on the machining efficiency.

**Figure 22:**
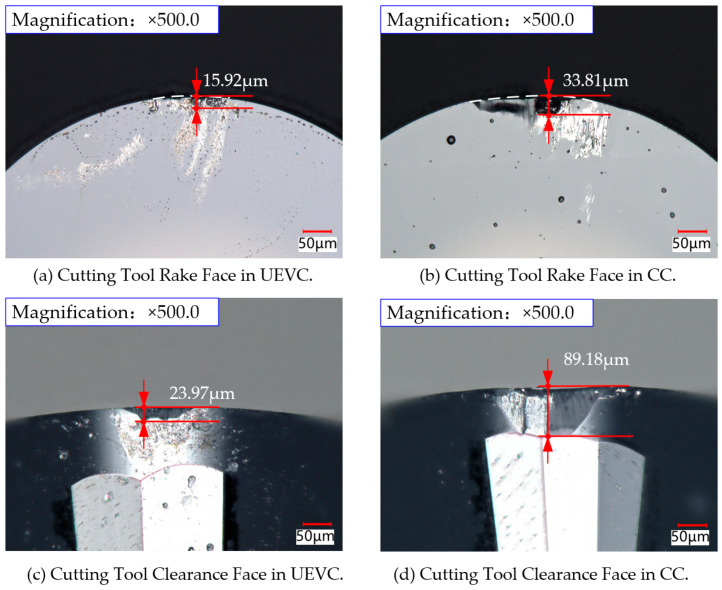
Tool wear under UEVC and CC.

Using an ultra-depth-of-field microscope, a comparative analysis of the tool wear in diamond tools during both conventional cutting and ultrasonic elliptical vibration cutting was conducted. The tool wear under these two cutting methods is shown in Figure 22. The experimental results indicate that in conventional cutting, built-up edge formation is more common at the tool tip, whereas this phenomenon is rarely observed during ultrasonic elliptical vibration cutting. The degree of tool wear on both the rake and flank faces is notably higher in conventional cutting compared to ultrasonic elliptical vibration cutting. Specifically, the flank wear of the tool in ultrasonic elliptical vibration cutting was 23.97 µm, compared to 89.18 µm in conventional cutting, representing a 73% reduction in wear. On the rake face, the wear with ultrasonic elliptical vibration cutting was 15.92 µm, while in conventional cutting, it was 33.81 µm, resulting in a 53% reduction in wear. These findings indicate that ultrasonic elliptical vibration cutting significantly reduces the tool wear during the machining of nickel-based superalloys.

The main reason for this improvement is that ultrasonic elliptical vibration cutting reduces the contact time between the tool and the workpiece and lowers the friction coefficient during contact, thereby effectively decreasing the occurrence of wear. Ultrasonic elliptical vibration cutting operates in a discontinuous cutting mode, characterized by short contact cycles between the tool and workpiece and an interrupted cutting process. This mode reduces the continuous contact time, lowers the friction and heat buildup between the tool and workpiece, and significantly reduces tool wear. Furthermore, the separation effect in discontinuous cutting helps reduce the adhesion between the tool and workpiece, inhibiting built-up edge formation. Since built-up edges typically accelerate tool wear, the reduction in this phenomenon during ultrasonic elliptical vibration cutting leads to a lower tool wear rate.

## 5. Conclusions

This study investigates the surface integrity of nickel-based superalloys under ultrasonic elliptical vibration cutting conditions. The influences of different process parameters on the surface roughness, residual stress, and microhardness of the workpieces were systematically investigated. Comparative experiments between ultrasonic elliptical vibration cutting and conventional cutting were conducted. The main conclusions can be drawn as follows:

(1) The surface roughness of nickel-based superalloys under ultrasonic elliptical vibration cutting increases with the increase in the ultrasonic amplitude, cutting depth, and feed rate. It shows a trend of first decreasing and then increasing with the increase in the cutting speed, and decreases with the increase in the tool tip radius. Choosing an appropriate cutting speed, feed rate, cutting depth, ultrasonic amplitude, and tool tip radius can reduce the surface roughness by 62%, 61%, 36%, 46%, and 52%, respectively.
(2) The residual compressive stress of the nickel-based superalloys under ultrasonic elliptical vibration cutting decreases with the increase in the cutting speed, while it increases with increased cutting depth and tool tip radius. It shows a trend of first increasing and then decreasing with the increase in ultrasonic amplitude. Choosing an appropriate cutting speed, cutting depth, ultrasonic amplitude, and tool tip radius can increase the residual compressive stress by 37%, 17%, 35%, and 32%, respectively.
(3) The microhardness of the nickel-based superalloys under ultrasonic elliptical vibration cutting increases with the increase in feed rate, cutting depth, and ultrasonic amplitude, and it first decreases and then increases with the increase in cutting speed. Choosing an appropriate cutting speed, feed rate, cutting depth, and ultrasonic amplitude during precision machining can increase the microhardness by 32%, 16%, 35%, and 35%, respectively.
(4) Compared with conventional cutting, ultrasonic elliptical vibration cutting has significant advantages in optimizing machining performance. At cutting depths of 2 µm, 3 µm, 4 µm, and 5 µm, the surface roughness decreased by about 19%, 27%, 12%, and 16%; the residual compressive stress increased by about 44%, 28%, 22%, and 11%; and the microhardness increased by 21%, 16%, 14%, and 10%, respectively. Meanwhile, the cutting tool wear reduced by approximately 53% under ultrasonic elliptical vibration cutting.

## Figures and Tables

**Table 1:** Main performance parameters of GH4169.

Element	Ni	Cr	Nb	Mo	Ti	Al	C	Si	Mn	Fe
*Wt* (%)	51.75	17	5.15	2.93	1.07	0.45	0.042	0.21	0.03	surplus

**Table 2:** Main chemical components GH4169.

Workpiece	Density *ρ* (kg/m^3^)	Hardness (HB)	Yield Strength *σ*_0.2_ (MPa)	Tensile Strength *σ_b_* (MPa)	Elongation *δ_s_* (%)	Shrinking Percentage *ψ* (%)
GH4169	8280	300	1260	1430	24	40

**Table 3:** Experimental parameters of GH4169 cutting.

No.	Cutting Speed v/(m/min)	Cutting Depth a_p_/(µm)	Feed Rate f/(µm/rev)	Ultrasonic Amplitude A, B/(µm)	Tool Nose Radius r_e_/(mm)
1	5	2	22	**A** = 7.0, **B** = 1.7	0.5
2	6	2	22	**A** = 7.0, **B** = 1.7	0.5
3	7	2	22	**A** = 7.0, **B** = 1.7	0.5
4	8	2	22	**A** = 7.0, **B** = 1.7	0.5
5	7	2	36	**A** = 7.0, **B** = 1.7	0.5
6	7	3	36	**A** = 7.0, **B** = 1.7	0.5
7	7	4	36	**A** = 7.0, **B** = 1.7	0.5
8	7	5	36	**A** = 7.0, **B** = 1.7	0.5
9	6	3.5	14	**A** = 7.0, **B** = 1.7	0.5
10	6	3.5	20	**A** = 7.0, **B** = 1.7	0.5
11	6	3.5	26	**A** = 7.0, **B** = 1.7	0.5
12	6	3.5	32	**A** = 7.0, **B** = 1.7	0.5
13	8	2	36	**A** = 3.9, **B** = 0.9	0.5
14	8	2	36	**A** = 6.0, **B** = 1.5	0.5
15	8	2	36	**A** = 8.0, **B** = 1.9	0.5
16	8	2	36	**A** = 8.9, **B** = 2.3	0.5
17	6	2.5	18	**A** = 7.0, **B** = 1.7	0.2
18	6	2.5	18	**A** = 7.0, **B** = 1.7	0.5
19	6	2.5	18	**A** = 7.0, **B** = 1.7	0.8

**Table 4:** Experimental parameters of UEVC and CC.

No.	Experimental Parameters				
	Cutting Speed v/(m/min)	Cutting Depth a_p_/(µm)	Feed Rate f/(µm/rev)	Ultrasonic Amplitudes A, B/(µm)	Tool Nose Radius r_e_/(mm)
UEVC	5	2, 3, 4, 5	22	A = 7.0, B = 1.7	0.5
CC	5	2, 3, 4, 5	22	0	0.5

## Data Availability

The original contributions presented in the study are included in the article, further inquiries can be directed to the corresponding author.
